# Complete genome analysis of a frog virus 3 (FV3) isolate and sequence comparison with isolates of differing levels of virulence

**DOI:** 10.1186/1743-422X-11-46

**Published:** 2014-03-12

**Authors:** Elizabeth A Morrison, Shawn Garner, Pierre Echaubard, David Lesbarrères, Christopher J Kyle, Craig R Brunetti

**Affiliations:** 1 Department of Biology, Trent University, 1600 East Bank Dr., Peterborough, Ontario K9J 7B8, Canada; 2 Current address: Department of Biology, Western University, 1151 Richmond Street, London, Ontario N6A 5B7, Canada; 3 Genetics and Ecology of Amphibians Research Group (GEARG), Department of Biology, Laurentian University, Sudbury, Ontario P3E 2C6, Canada

**Keywords:** FV3, 454 GS-FLX technology, Viral isolates, Genetic variation, Virulence

## Abstract

**Background:**

Frog virus 3 (FV3) is the type species of the genus *Ranavirus*, and in the past few decades, FV3 infections have resulted in considerable morbidity and mortality in a range of wild and cultivated amphibian species in the Americas, Europe, and Asia. The reasons for the pathogenicity of FV3 are not well understood.

**Findings:**

We investigated three FV3 isolates designated SSME, wt-FV3, and aza-C^r^, and reported that our wt-FV3 and aza-C^r^ strains showed similar levels of virulence, while SSME was the least virulent in an *in vivo* study with *Lithiobates pipiens* tadpoles. Using 454 GS-FLX sequencing technology, we sequenced SSME and compared it to the published wt-FV3 genome. SSME had multiple amino acid deletions in ORFs 49/50L, 65L, 66L, and 87L, which may explain its reduced virulence. We also investigated repeat regions and found that repeat copy number differed between isolates, with only one group of 3 isolates and 1 pair of isolates being identical at all 3 locations.

**Conclusions:**

In this study we have shown that genetic variability is present between closely related FV3 isolates, both in terms of deletions/insertions, and even more so at select repeat locations. These genomic areas with deletions/insertions may represent regions that affect virulence, and therefore require investigation. Furthermore, we have identified repeat regions that may prove useful in future phylogeographical tracking and identification of ranaviral strains across different environmental regions.

## Background

Global amphibian populations have declined considerably in recent years, in part due to habitat fragmentation, pollution, and the chytrid fungus *Batrachochytrium dendrobatidis*[1,2]. More recently, certain members of the family *Iridoviridae* have also been associated with amphibian decline. The family *Iridoviridae* is comprised of large, cytoplasmic, double stranded DNA viruses with icosahedral capsids [3], and is divided into five genera: *Iridovirus*, *Chloriridovirus*, *Lymphocystivirus*, *Megalocytivirus*, and *Ranavirus*[4]. Specifically linked to amphibian mortality in this family are infectious diseases caused by members of the genus *Ranavirus*. In past research, ranaviruses received little attention as most infections were deemed subclinical; however, recent ranavirus infections have resulted in considerable morbidity and mortality in a range of wild and cultivated amphibian species in the Americas, Europe, and Asia [5-8]. It has been reported that 43% of known amphibian die-offs in the USA from 2000 to 2005 were due to ranaviruses [9], and that from 1996–2001 ranaviruses were isolated from most of the amphibian mortality events in North America [10]. Detection of these outbreaks could be due to better surveillance, increased environmental awareness, the mutation of viral species creating highly pathogenic strains, or environmental changes resulting in host immune suppression [11]. Ranaviruses have become a significant cause of death and disease in amphibians, and thus investigation into these viruses is warranted from a virological, commercial, and ecological standpoint [11].

Frog virus 3 (FV3) is the type species of the genus *Ranavirus*[4]. FV3′s genome is 105,903 base pairs (bp) comprised of 98 open reading frames (ORFs) [12]. Depending on factors such as strain virulence and host immune response, infection with FV3 may or may not lead to mortality. However, in susceptible amphibians, FV3′s necrotic and apoptotic effects cause systemic, chronic cell death in multiple internal organs, resulting in death of the host within a few days to several weeks [2,13,14]. FV3 infection is also marked by cutaneous signs, including ulceration of the skin, and erythema and swelling of the limbs and body. In fatal cases, intracoelomic lesions are often present, including haemorrhages of the kidneys and reproductive organs, and pale, swollen livers [15]. While our understanding of ranavirus pathogenicity has improved over the last decade, there is still a need for the research community to more fully describe the determinants of virulence variation. Elucidation of this area will likely heavily rely on the genetic analysis and comparison of ranaviruses that differ in host range and virulence.

Genetic comparison of related DNA viruses has proven to be an important tool in classifying viral strains and understanding the epidemiology and evolution of different genotypes [16]. By analyzing genetic differences, researchers can link clinically significant alterations with molecular changes, and better understand viral origins and evolution. Increasingly, new viral strains are being identified based on the systematic analysis of sequence data, including short amino acid insertions, translational stop codons, and single amino acid deletions [17]. Analysis of viral isolates has led to the discovery of new viral genotypes, as well as better understanding of the functional genetic differences among strains [17]. For instance, the entire genome of a virulent strain of duck entiritis virus was recently sequenced and compared to the genomes of an attenuated strain and another virulent strain [18]. The results indicated several nucleotide insertions/deletions and frame-shift mutations effecting ORF initiation or termination [18]. These findings allowed the researchers to identify possible virulence factors and provided information on ORFs that are changed during serial passage.

In addition to nucleotide insertions/deletions, variation between viral genomes may occur at repeat regions. Eaton et al. [19] identified repetitive sequences in the genomes of various ranaviruses with high copy number variation. Repetitive sequences are commonly classified into one of three groups: macro, mini, or micro satellites [20]. The repeats that we will examine in this study contain less than 400 bp comprised of 9-19 bp repeating units. Thus, we suggest that these repeats are some variation between micro and mini satellites, and for the purposes of our analysis, will be referred to as short tandem repeats (STRs). A selection of these repeat regions will be used to analyze FV3 isolates in order to further investigate the fine scale, genetic differences present in variable regions.

Past studies on the genetic variation between DNA viral strains have allowed for the detection of minute genetic changes that would otherwise have gone unnoticed. These kinds of changes have proved useful in explaining phenotypic differences and evolutionary histories. The purpose of the present investigation is to narrow our focus even further by comparing the genomes of closely related FV3 isolates, including those with varying levels of virulence. This will be done in an attempt to explore the genetic diversity present in strains of FV3, with the ultimate goal of further elucidating the possible genetic basis behind FV3′s unpredictable infectious behaviour.

## Results

### FV3 strains differ in virulence during in vivo infection

**Figure 1:**
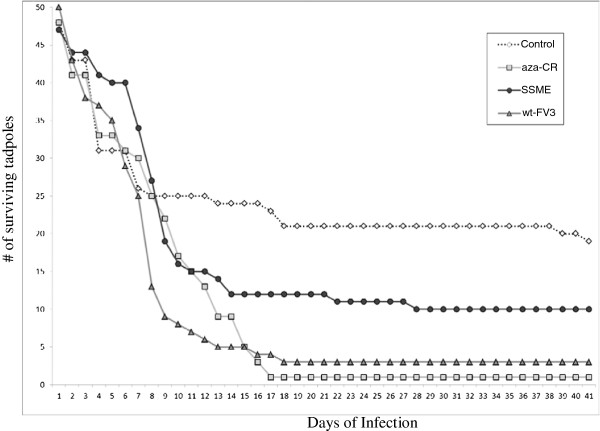
**Variable virulence of three FV3 strains during tadpole infection.** Survival analysis of *L. pipiens* tadpoles over 41 days of infection with aza-C^r^ (square), SSME (circle), wt-FV3 (triangle), and Control (diamond). Tadpoles were exposed to FV3 strains for 12 hours, and then along with the virus-contaminated water were transferred to a tank of dechlorinated water for the remainder of the experiment. Survival analysis and failure time analysis was done following the Kaplan & Meier product limit method associated with Chi square and Gehan’s Wilcoxon tests [37].

In order to determine if FV3 strains SSME, wt-FV3, and aza-C^r^ induced different degrees of infection, *L. pipiens* tadpoles were exposed to each strain. Tadpoles were exposed to FV3-infected water for 12 h before being transferred to FV3-free water for the remainder of the experiment. The tadpoles were monitored for 41 days, at which time all tadpoles had either died or reached metamorphosis. The results of the survival analysis revealed significant differences in strain effect on tadpole mortality rates (X^2^ = 21.3, p < 0.01). Although deaths triggered by wt-FV3, aza-C^r^, and SSME stocks peaked between 15 and 18 days post infection, overall mortality was higher following infection with wt-FV3 and aza-C^r^ virus (>96%) than with SSME (84%). Wt-FV3 induced a mortality of 97%, and aza-C^r^ a mortality rate of 96% (Figure 1). In contrast, SSME infected tadpoles reached a mortality plateau after 28 days, and showed a mortality rate of 84%. The control tadpoles had the lowest mortality, with a rate of 66% after 41 days (Figure 1). These data demonstrate that the closely related FV3 strains, wt-FV3 and aza-C^r^[21,22], are associated with higher mortality than SSME (Figure 1). Of concern was the high mortality seen even in control tadpoles (Figure 1). We did confirm that the control tadpoles, both those that survived and those that died, were ranavirus negative (data not shown). This mortality could be the result of overcrowding of tadpoles during infection. In addition, this mortality is occurring during the first few days of the experiment at a time when the tadpoles are very small and very weak, very sensitive to stressful conditions. At the beginning of the experiment, the tadpoles are transferred from tank to tank that may account for the high mortality rate early on in the experiment or may have resulted in high mortality of a sublethal viral dose.

### Genomic sequencing of SSME

In order to better understand the possible genetic basis of phenotypic variation among strains, we sequenced the SSME strain using 454 GS-FLX technology. Our sequenced genome showed high similarity to the published FV3 sequence (wt-FV3) [12], with a sequence identity of 98.79% and average genome sequencing coverage of 51x. Results revealed that SSME differed from the wt-FV3 genome length of 105,903 bps and ORF number of 98; instead, SSME had a length of 105,070 bps, and a total of 95 predicted ORFs (Table 1). Despite high overall sequence identity between SSME and FV3, marked differences were noted in several regions of the genome (Table 2).

**Table 1:** Description of nucleotide start/stop locations and amino acid length of ORFs in SSME, along with predicted functions

	**Start/stop (#aa)**	
**ORF**	**SSME**	**Predicted function/conserved domains**
1R	272-1042	Replication factor &/or DNA packing protein
	255aa	
2L	1649-2629	Myristylated membrane protein, DUF230 poxvirus protein, TM*
	325aa	
3R	3436-4752	IIV6 ORF 229L, SP
	437aa	
4R	4793-4975	TM
	59aa	
5R	5408-6022	FPV ORF 250, US22, herpes virus early nuclear protein
	203aa	
6R	6025-6252	SP*
	74aa	
7R	7043-7429	SP
	127aa	
8R	7521-11402	DNA-dependent RNA polymerase largest subunit
	1292aa	
9L	11771-14617	NTPase, SNF2 family, N-terminal, helicases C-terminal, DEAD/H helicases
	947aa	
10R	14633-15046	TM
	136aa	
11R	15396-15608	TM
	69aa	
12L	15674-16567	Unknown protein, SP
	296aa	
13R	17108-17314	SP
	67aa	
14R	17329-17688	Unknown protein, SP
	118aa	
15R	17784-18752	AAA-ATPase, poxvirus A32
	321aa	
16R	19032-19859	Integrase homologue
	274aa	
17L	20100-21608	SP
	501aa	
18 L	21645-21881	TM
	77aa	
19R	21933-24515	Similar to LCDV1 or f10L conserved uncharacterized protein, SP
	859aa	
20R	24562-25008	Unknown protein, TM
	147aa	
21L	25231-25890	ISKNV ORF 56L-like protein
	218aa	
22R	26020-28941	D5 family NTPase, ATPase
	972aa	
23R	29319-30467	SP
	381aa	
24Rº	30877-31947	SP
	355aa	
25R	32141-32929	P31K protein
	261aa	
26R	32996-33226	Truncated elf-2α homologue
	75aa	
27R	33757-36669	Tyrosine kinase, CAP 10, putative lypopolysaccharide modifying enzyme
	969aa	
28R	36718-37206	Unknown protein, SP
	161aa	
29L	37385-37681	SP
	97aa	
30R	37883-38035	TM
	49aa	
31R	38097-38516	SP
	138aa	
32R	38566-40458	Neurofilament triplet H1 protein
	629aa	
33R	40541-40732	Unknown protein, TM
	62aa	
34R	40875-41195	Human parainfluenza virus 1L like protein, TM
	105aa	
35L	41287-41748	SP
	152aa	
36L	41761-42384	SP
	206aa	
37R	42778-43413	NIF/NLI interacting factor
	210aa	
38R	43554-45251	Ribonucleoside diphosphate reductase alpha subunit barrel domain
	564aa	
39R	45357-45707	Hydrolase of the metallo-beta-lactamase superfamily
	115aa	
40R	45795-46343	TM
	181aa	
41R	46725-50222	RRV ORF-2-like protein, SP
	1164aa	
42L	50718-50975	SP
	84aa	
43R	50974-51491	TM
	171aa	
44R	51512-51697	SP
	60aa	
45L	51973-52383	LCDV1 ORF-88-like protein, SP
	135aa	
46L§	52437-53000	RGI 47L-like protein, SSTIV 049L-like protein, Neurofilament triplet H1-like protein, microneme/rhoptry antigen
	186aa	
47L	53125-53541	SP
	137aa	
48L	53544-53795	SP
	82aa	
49/50L¶	53904-55451	LCDV1 ORF 58-like protein, RGI ORF 50L-like protein, SSTIV ORF 052-like protein, SAP DNA binding domain
	514aa	
51R	55531-57216	Unknown, SP
	560aa	
52L	57473-58540	3-beta-hydroxy-delta 5-C27 steroid oxidoreductase-like protein, TM
	354aa	
53R	58878-60446	LCDV1 ORF-20-like protein, SP
	521aa	
54L	60677-60907	Nuclear calmodulin-binding protein
	75aa	
55L	60945-62240	Helicase-like protein
	430aa	
55R	61089-62228	FV3 40-kDa protein, SP
	378aa	
56R	62328-62765	SP
	144aa	
57R	62879-64375	Phosphotransferase, S-TKc, Serine/Threonine protein kinase
	497aa	
58R	64700-65413	SP
	236aa	
59L	65964-67022	RGV 9807 unknown protein, SP
	351aa	
60R	67184-70225	DNA polymerase-like protein, DNA polymerase family B exonuclease
	1012aa	
61L	70234-70416	SP
	59aa	
62L	70859-74524	DNA-directed RNA polymerase II second largest subunit RNA polymerase domain 6, 7, 3, 2 beta subunit
	1220aa	
63R	74903-75397	dUTPase-like protein
	163aa	
64R	75522-75809	Interleukin-1 beta convertase precursor, Caspase-recruitment domain/DEATH
	94aa	
65L^#^	N/A	SP
66L^#^	76106-76157	SP
	16aa	
67L	76212-77375	Ribonucleoside-reductase diphosphate beta subunit-like protein
	386aa	
68R	77657-77944	SP
	94aa	
69R	78080-78346	Unknown protein, TM
	87aa	
70R	78364-78738	SP
	123aa	
71R	78778-79011	SP
	76aa	
72L	79068-79784	SP
	238aa	
73L	80232-81206	NTPase/helicase-like protein
	323aa	
74L	81381-82493	SP
	369aa	
75L	82525-82779	LITAF/PIG7 possible membrane associated motif in LPS-induced tumor necrosis factor alpha factor, TM
	83aa	
76R	82842-83063	SP
	72aa	
77L	83060-83407	LCDV ORF 2-like protein, SP
	114aa	
78L	84007-84645	SP
	211aa	
79R	84781-86499	ATPase-dependent protease
	571aa	
80L	87122-88237	Ribonuclease III-like protein
	370aa	
81R	88293-88571	Transcription elongation Factor SII, C2C2 zinc finger
	91aa	
82R	88700-89173	Immediate-early protein ICP-18
	156aa	
83R	89623-90267	Cytosine DNA methyl-transferase
	213aa	
84R	90652-91389	LCDV 1-like proliferating cell nuclear antigen, SP
	244aa	
85R	91464-92051	Deoxynucleoside kinase
	194aa	
86L	92441-92626	SP
	60aa	
87L**	92979-94778	Unknown protein, SP
	598aa	
88R	94811-95263	Evrl-air-augmenter of liver regeneration
	149aa	
89R	95331-96497	SP
	388aa	
90R	96590-97981	Major capsid protein
	462aa	
91R	98105-99292	Immediate-early protein ICP-46
	394aa	
92R	99627-99872	SP
	80aa	
93L	100054-100221	SP
	54aa	
94L	100331-100798	Regina ranavirus P8.141 C-like protein, TM
	154aa	
95R	100891-101982	DNA repair protein RAD2, Xeroderma pigmentosum G N-region, Helix-hairpin-helix, Class 2 (Pol I) family
	362aa	
96R	102784-103455	Unknown protein, SP
	222aa	
97R	103538-103951	Myeloid cell leukemia protein, MCL-1, TM
	136aa	
98R	104649-104849	SP
	65aa	

*Note: SP-surface protein, TM-transmembrane domain.

ºLoss of original, predicted start codon in SSME; new start codon 27 bp downstream.

§Loss of original, predicted stop codon in SSME; new stop codon 319 bp upstream.

¶39 bp deletion in original 50L ORF of SSME. Loss of ORF 50L’s original, predicted stop codon in SSME; ORFs 49L and 50L are now combined as 49/50L.

#Deletion of 65L ORF and majority of 66L ORFs in SSME.

**18 bp deletion in 87L ORF of SSME.

**Table 2:** Genetic variation present within the SSME genome as compared to the wt-FV3 reference genome

**Region**	**Change**	**Affect**
21,933-24,515 bp	24 bp substitutions	24aa substitutions & 9aa insertion in 19R
	27 bp insertion	
30,851 bp	1 bp substitution	Loss of a start codon in 24R; new start codon is 27 bp downstream, thus shortening ORF by 9aa
51,102 bp	1 bp deletion	Frameshift mutation in 43R
52,769 bp	1 bp deletion	Frameshift mutation and loss of original stop codon in 46L; new stop codon is 319 bp upstream, thus lengthening 46L
54,805 bp	1 bp deletion	Loss of original stop codon in 50L, leading to the combination of ORFs 49L and 50L
54,938 bp	39 bp deletion	13aa deletion in 49/50L
76,105 bp	757 bp deletion	Deletion of all of 65L and the majority of 66L
94,712 bp	18 bp deletion	6aa deletion in 87L
10,4050 bp	67 bp deletion	67 bp deletion in non-coding region between 97R and 98R

Genome region involved, nucleotide change, and end affect of the variation is presented.

### Gene variation between ranaviruses

In order to investigate the possible genetic variation within coding regions and regions of high variability between closely related viral isolates, we compared our sequenced SSME genome with four published ranavirus genomes. The genomes compared included: SSME, wt-FV3 [17], rana grylio iridovirus (RGV) [23], soft-shelled turtle iridovirus (SSTIV) [24], and tiger frog virus (TFV) [25]. We began by focusing our analysis on predicted coding regions, as variation in these areas may have consequences in pathogenicity or the kind of disease a virus causes.

**Figure 2:**
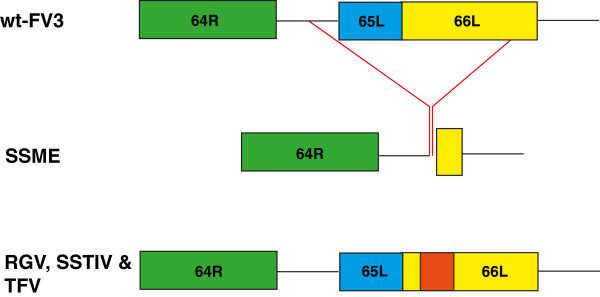
**65L & 66L deletion in SSME.** Alignment of the 64R-66L region across ranaviruses. A 757 bp deletion is present in the SSME genome, spanning from 76,113-76,869 bp in wt-FV3, deleting all of 65L and most of 66L. A 139 bp insertion is also present in the 66L homologous regions of RGV, SSTIV, and TFV genomes.

Upon analysis of selected ranaviral genomes, a 757 bp deletion was discovered in SSME, deleting the entire 65L coding region and the majority of the 66L coding region (Figure 2). This deletion was not present in wt-FV3 or in related ranaviruses RGV, SSTIV, and TFV. However, sequence alignments indicated reorganization of 66L in RGV, SSTIV, and TFV, with a 139 bp insertion found in their corresponding 66L regions (Figure 2). Therefore, there are at least 3 different genomic presentations of this region in ranaviral genomes, making it highly variable among isolates.

**Figure 3:**
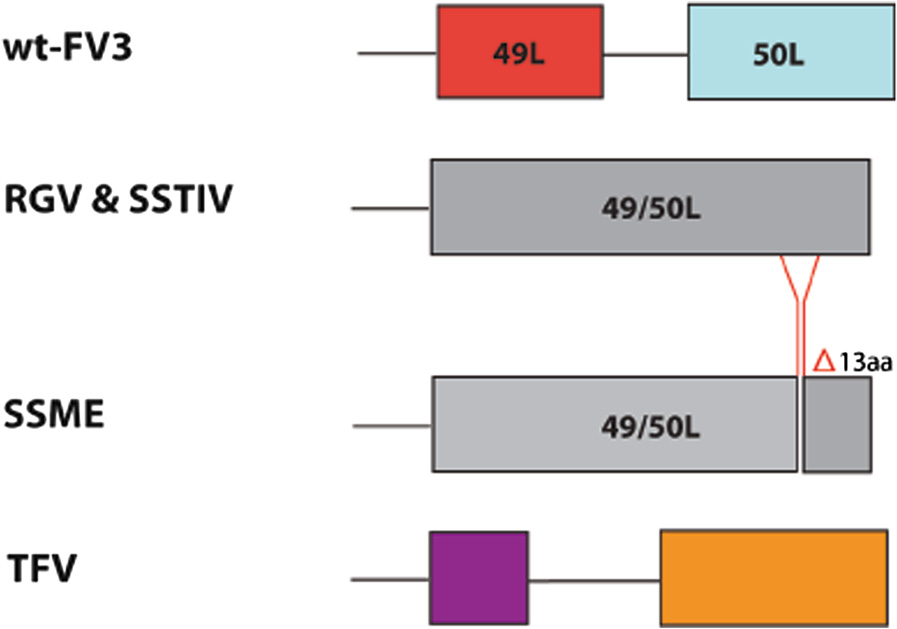
**Variation in ORF 49/50L.** Sequence alignment of the 49/50L region across ranaviruses. A 39 bp deletion is present in the SSME genome, spanning from 54,909-54,947 bp in wt-FV3. A single nucleotide deletion is present in SSME, RGV, and SSTIV, leading to the loss of 50L’s original stop codon and the merger of 49L and 50L into one ORF. Different colours are used to represent the portions of TFV’s ORFs that are homologous to parts of 49L and 50L respectively.

Another coding region demonstrating variation was 50L. An in frame deletion of 13 amino acids was found in 50L when compared to wt-FV3, present only in the SSME genome (Figure 3). In addition, 50L had a single nucleotide deletion resulting in a frameshift mutation in SSME, RGV, and SSTIV when compared to wt-FV3. This frameshift mutation resulted in the loss of 50L’s stop codon, which led to the combination of 49L and 50L into a single ORF. These two ORFs merged in frame, creating 49/50L. In contrast, TFV did not have this single nucleotide deletion, nor did it display the merger of its 49L and 50L equivalent ORFs (Figure 3). This is consistent with TFV being the most divergent virus of the group.

**Figure 4:**
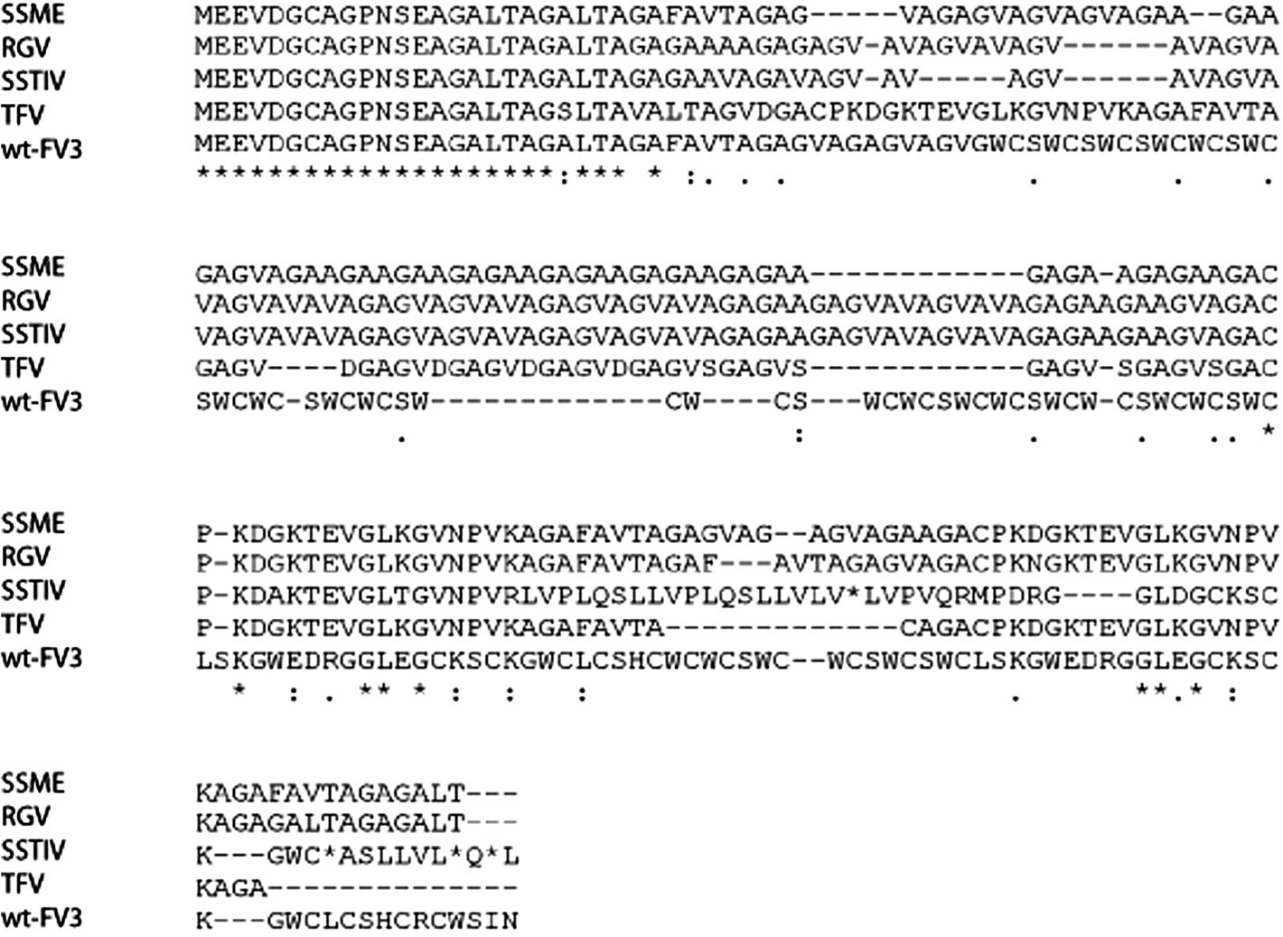
**Variation in 43R ORF.** Amino acid sequence alignment of the 43R ORF in ranaviruses. A single nucleotide deletion in SSME, RGV, SSTIV, and TFV led to frameshift mutations, causing significant amino acid variability amongst genomes.

Further ranaviral gene variation was discovered in 43R in the form of a single nucleotide deletion, resulting in a frameshift mutation. Similar deletions were found in SSME, RGV, SSTIV, and TFV, leading to pronounced differences in amino acid sequences when compared to wt-FV3 (Figure 4). It is worth noting that although SSME, RGV, and SSTIV share the same frameshift mutation the genes are not identical as there are several single amino acid changes that lead to modest variation (Figure 4). In contrast, despite having a similar nucleotide deletion, TFV’s proposed amino acid sequence was markedly different from other analyzed ranaviruses. Another single nucleotide deletion was found in 46L (Tables 1 and 2), which led to the loss of the original stop codon and the extension of the ORF by 319 bps (Tables 1 and 2). This was found in all genomes other than TFV and wt-FV3. These data demonstrate that even between very similar viral genomes, there is considerable gene variation, and that when viruses differ in pathogenicity, it could be related to variation in coding regions.

### Repeat regions can vary in copy number between viral isolates

Genomic sequencing, and subsequent analysis, of SSME in comparison to wt-FV3, provided a general overview of the genetic variation between closely related FV3 isolates, particularly in coding regions. We then decided to explore possible genetic variation in highly variable repeat regions, and thus define these genetic regions with greater inter-strain variability. To achieve this, we chose three repeat regions (suspected to be polymorphic in copy number) to investigate. These regions included: Region 1 (22,499-22,574 bps); Region 2 (52,443-52,747 bps); and Region 3 (54,948-54,986 bps), based on the wt-FV3 sequence. We performed a repeat analysis on our sequenced SSME genome that we had previously evaluated for coding region variability, and reference genomes RGV, SSTIV, and TFV. We also decided to analyze the repeat regions of wt-FV3 and aza-C^r^ in order to check for repeat number stability between the two strains over the multiple viral passages they have undergone since the creation of aza-C^r^ in 1987 [22]. In addition, we expanded our analysis to include 6 environmental samples isolated from the same waterway in Manitoulin, Kagawong, ON, Canada. These isolates were designated as: E3, E4, E5, F4, F6, and G4; a lab FV3 sample (ATCC® VR-567™) was also used [26].

**Figure 5:**
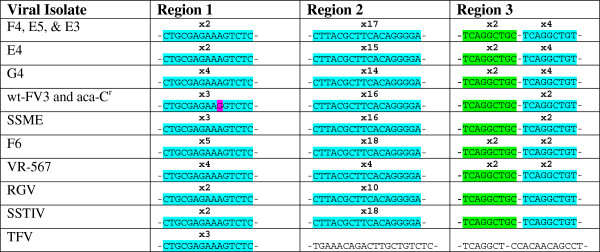
**STR variation summary.** Short tandem repeats (STRs) vary between viral isolates at three tested locations. Results reveal three isolates (F4, E5, and E3), and two isolates (wt-FV3 and aza-C^r^), that have the same STR copy number at each location. Areas highlighted in blue are full repeats, with their coinciding copy number above. Areas highlighted in green represent a sequence that may/may not repeat but is very similar to the STR that follows it in the genome, and areas highlighted in pink represent nucleotides that differ from the STR found in the majority of ranaviruses analyzed.

The information acquired through analyzing these repeat regions indicated that variation in repeat copy number is present between ranaviral isolates, including those from one geographic location (Figure 5). In the first region, repeat copy number ranged from 2–5 copies, with 2 copies being the most common and 5 copies being unique to isolate F6. Wt-FV3 and aza-C^r^ shared a distinctive repeat sequence, containing a G rather than an A at the 9^th^ nucleotide position (Figure 5). Region 2 proved to be more variable than region one, with repeat copy number ranging from 4–18 copies, while Region 3 was found to consist of two repeat sequences. These Region 3 repeats varied in copy number between isolates, with a maximum copy number of 4. However, in some instances, the first sequence of nucleotides characteristic of this region did not repeat (Figure 5).

Although copy number variation was present amongst isolates, when samples were compared across all three repeat regions, some were found to be identical; such isolates included F4, E5, and E3 (Figure 5), which were isolated from the same geographic region. In addition, isolates wt-FV3 and aza-C^r^ were also found to be identical across repeat regions (Figure 5), which is consistent with their common origin [22]. This data taken as a whole suggests that viral isolates, even those from one geographic location, can display both variation and identical patterns across repetitive regions of the genome. Thus, these repeats could act as a fingerprint to discriminate between viral isolates.

## Discussion

Our analysis of related ranaviruses presents a novel approach to genomic comparison that differs from other studies. We analyzed the genomes of closely related isolates of FV3 through 454 GS-FLX technology and STR comparison. The scale (fully sequenced genomes), and the nature of comparison (using viral isolates of FV3), set our investigation apart from past studies. We found that the 3 strains we examined displayed slightly different levels of virulence during *in vivo* studies. By sequencing the one genome yet to be sequenced, we were able to highlight areas that may be important in generating infectious phenotypes of FV3. This kind of analysis has never been done for FV3 or related ranaviruses: thus, it provides greater insight into the genetic variation among these closely related DNA viruses and the possible genetic basis of ranaviral virulence.

Our results demonstrate that genetic variation is present between closely related FV3 isolates in both coding and non-coding regions. The SSME genome was sequenced and compared to the published wt-FV3 genome, along with related ranaviral genomes RGV, SSTIV, and TFV. Comparisons revealed that SSME was divergent from wt-FV3, and aza-C^r^. This variation could be due to the fact that SSME was isolated from a spotted salamander rather than anurans, and so the strain may have evolved in order for it to better adapt to its novel host, as is seen during serial passage [27]. For example, the pathogenicity of Dengue virus was altered by serial passaging Dengue virus 27 times which resulted in 25 nucleotide changes between 2 strains [27]. This finding is interesting given the fact that our results also showed that SSME was the least virulent of our 3 strains during an *in vivo* study in anurans (Figure 1). Thus, genetic mutations observed in SSME, such as deletions in ORFs 65L, 66L and 49/50L, could have had an effect on strain virulence in anuran hosts. Also, past sequencing results [12] as well as our own genomic sequencing of wt-FV3 and aza-C^r^ genomes revealed only 13 nucleotide differences (data not shown) between the strains, supporting our *in vivo* finding that the two strains cause essentially the same amount of tadpole mortality (97% and 96% respectively).

One of the most significant genetic variations found in SSME was a 757 bp deletion that deleted all of the 65L coding region and most of the 66L coding region (Figure 2). Among other ranaviruses, the functions of these genes have not been determined. Further sequencing of ranaviruses has shown that 65L is present in both RGV and SSTIV as 69L and 68L respectively [23,28], while this form of 66L appears to be unique to FV3, as other ranaviruses have a 139 nucleotide insertion in their related 66L regions (Figure 2). Another genetic variation specific to SSME was a 13 amino acid deletion in ORF 50L (Figure 3). Interestingly, a second variation was found in this area in the form of single nucleotide deletion. This deletion lead to the merger of ORFs 49L and 50L in all examined ranaviruses other than TFV and wt-FV3 (Figure 3). In terms of function, 49L has multiple SAP motifs, which are DNA/RNA binding domains predicted to be involved in chromosomal organization and DNA replication [29]. Thus, this new 49/50L ORF may function in viral replication.

Single nucleotide deletions were found in multiple ranaviruses within the 43R genes (Figure 4) and 46L (Tables 1 and 2) genes. In 43R, the deletion was present in all ranaviruses analyzed other than wt-FV3, and resulted in a frameshift mutation (Figure 4). In 46L, the original stop codon was lost, leading to the extension of the ORF by 319 bps (Tables 1 and 2). This was found in all genomes other than TFV and wt-FV3. Supposedly, 46L encodes for a neurofilament triplet H1-like protein [12]. However, the extended version of 46L that we discovered has a putative conserved domain known as a microneme/rhoptry antigen in the area previously thought to be non-coding. Micronemes and rhoptries are organelles possessed by Apicomplexa protozoans that secrete proteins involved in parasite entry into a host cell, specifically possessing protein-binding motifs that recognize ligands on the host cell surface [30]. Although usually associated with protozoan parasites, these microneme/rhoptry antigens found in 46L could give further indication as to 46L’s function.

The changes present in 43R, 46L, 49/50L, 65L, and 66L represent the main variable regions amongst the related ranaviral isolates we analyzed. This suggests that these are changeable areas across ranaviruses, and could be used in the future to help explain variable infectious phenotype. Moreover, multiple amino acid deletions present in 65L, 66L, and 49/50L, were limited to the SSME strain, which displayed the lowest level of virulence during tadpole infection. Thus, changes that are unique to SSME may present areas of the genome that are particularly effective in viral attenuation, specifically in an alternative host. As FV3 mortality and morbidity continues to worsen and fluctuate across environmental regions, examination of these genomic areas may prove useful as an initial way to investigate the genetic basis behind infective changes [6]. Further research could be used to explain variations in the virulence of different FV3-like isolates.

As we had already identified variation in the coding regions of closely related FV3 isolates, we decided to further our understanding of variation within highly variable sites by investigating 3 recently identified STR regions [19]. These are known to be variable areas: for instance, although FV3 and SSTIV share 99% genome sequence identity, they share only approximately 50% of repeats in common [19]. Thus, we predicted that these repeat regions would have greater inter-strain variability that would provide useful information when trying to understand overall genetic variability between ranaviral isolates. In order to test this prediction, we analyzed our sequenced SSME genome and reference ranaviral genomes, along with 6 environmental samples isolated from the same waterway. We also sequenced wt-FV3 and aza-C^r^ to check for repeat number stability across their past viral passages. Analysis revealed that repeat copy number was variable between isolates, even between those from the same geographic location, but that there was some conservation (Figure 5). Specifically, wt-FV3 and aza-C^r^ were identical, and samples F4, E5, and E3 were also identical at all three regions (Figure 5); this finding was not surprising given that aza-C^r^ is the result of the wt-FV3 strain treated with azacytidine and does not necessarily represent a strain with a separate evolutionary history [22]. It also implies stability in the repeat regions, as these regions have not changed between the two strains since their initial separation. However, the finding that viral isolates from the same geographical area have variability may limit the use of STRs as a geographic marker. This STR analysis allowed us to better quantify the small scale genetic variation that is present in highly variable genetic sites amongst FV3 isolates, thus furthering our understanding of genetic variation beyond coding regions.

The STR analysis we performed in our study has the potential to contribute to our understanding of FV3 tracking and strain designation. Surveillance and phylogeographical analysis of FV3 are pivotal in understanding how the pathogen varies between different habitat sites and amphibian species, as well as for revealing possible sources of a disease outbreak [31]. It can also have direct effects on conservation by aiding in strategy development to minimize die-offs in high-risk areas, and in creating vaccines through knowledge of the FV3 genome itself [14,31]. However, exact taxonomic identification of viruses in amphibian populations has been difficult given the lack of detailed molecular data on FV3 and other ranaviruses. The methodology used to classify these viruses in the past has been through comparing the major capsid protein (MCP) of different viral isolates [3]. However, the use of the MCP as a tool to distinguish between different ranaviruses, as well as between different strains of the same virus, has been called under scrutiny [24,32,33]. Thus, there is a need to develop new methods of strain tracking for ranaviral isolates.

The use of STRs for ranavirus strain identification has precedence in other virus studies. In one such study, 12 isolates of human cytomegalovirus (HCMV) were isolated from various individuals infected with the virus. The isolates were then tested for variable repeats in 24 polymorphic regions, and based on this analysis, each viral isolate was designated as an individual strain of HCMV [34]. Many of the HCMV repeats used in this study were found in non-coding regions of the genome, similarly to the ones used in our study. The study suggested that these changes in repeats are evolutionarily neutral and so appropriate for strain identification, not only in HCMV, but in other similar, large genome DNA viruses [34].

Other studies have used coding instead of non-coding repeat regions to identify viral strains [35,36]. In our study, Region 1 is found in the 19R ORF, unlike the non-coding areas of Regions 2 and 3. There are many examples of functional microsatellites that are known to affect viral characteristics based on copy number, including hepatitis C virus and vesicular stomatitis virus [35,36]. Therefore, in addition to being potentially useful in viral tracking, STRs from the Region 1 coding region may have functional significance in FV3.

## Conclusions

In this study we have been able to uncover fine scale genetic variation between closely related ranaviral isolates that have different levels of virulence. We have shown that substantial genetic variability is present between closely related FV3 isolates, both in terms of deletions/insertions, and even more so at select STR locations. These genomic areas with deletions/insertions may present regions that affect viral infectious phenotype, and therefore require investigation. Furthermore, we have identified STR regions that may prove useful in future phylogeographical tracking and identification of ranaviral strains across different environmental regions. As FV3 leads to more unexplained lethal infections in amphibian populations, studies such as this are necessary. The genetic insight that they provide into ranaviral genomes will prove invaluable when seeking to explain the variability of FV3 infectious phenotype in wild populations, and in preventing the devastation that ensues.

## Material and methods

### Reagents, viruses

FV3 isolate SSME, wt-FV3, and aza-C^r^, were analyzed in this study and were provided by Professor Gregory Chinchar from the University of Mississipi Medical Center (Jackson, MS, USA). SSME was isolated from a wild, spotted salamander population in Maine, USA, while aza-C^r^ was derived from the laboratory wt-FV3 strain through selection with azacytidine [22]. Both wt-FV3 and aza-C^r^ have been previously sequenced and found to be identical, with wt-FV3′s nucleotide sequence deposited into GenBank [12]. Amphibian renal cells (A6 cells) were supplied by Niels Bols of the University of Waterloo and maintained in Leibovitz’s L-15 media (Invitrogen, Burlington, ON) supplemented with 10% fetal bovine serum (FBS; Invitrogen, Burlington, ON), penicillin (100 U/ml), and streptomycin (100 μg/ml).

### Infection of tadpoles with FV3

In order to monitor survival of FV3 infected tadpoles, we obtained *Lithiobates pipiens* tadpoles, approximately Gosner stage 25 [37], from the Environment Canada Atlantic Laboratory for Environmental Testing in Moncton, NB., courtesy of Paula Jackman. The animals were received two weeks prior the beginning of the experiment and were kept in 20 L tanks filled with 10 L of aged clean dechlorinated water. Each treatment group was done as 2 replicates of 25 tadpoles per treatment. Groups tadpoles were placed in dechlorinated water, with the host density (number of tadpoles per volume of water) adjusted to 1 tadpole per 250 mL to avoid any effect of density on tadpole development [38]. To infect, 25 tadpoles were placed in 50 mL of infected water containing 10,000 pfu/mL of a FV3 strain (SSME, wt-FV3, and aza-C^r^)*.* According to past experiments, such concentration is known to induce sublethal effects in these laboratory conditions [38]. Control individuals were placed within 50 ml of FV3-free water. The tadpoles were left within the infected solution overnight (12 hours) tadpoles were then transferred together with the contaminated water in 2 L plastic containers filled with 1 L of dechlorinated water (aged for three days) for the rest of the experiment. Containers were held in a climatic chamber (Thermo Incubator Model 3740) where the temperature was set to remain at 22°C with a 12 h:12 h dark:light cycle. Tadpoles were fed on a weekly basis after the water was changed with standard tadpole food (Carolina Biological Supply Company, Burlington, NC) at 30 mg/tadpole for week 1, 60 mg/tadpole for week 2, and 120 mg/tadpole for week 3 until the end of the experiment [38]. Starting on week 3 the water in each tank was replaced once a week with clean dechlorinated aged (24 h) water. As a result, exposed tadpoles were held in virus-containing water for 3 weeks, a period which is long enough for tadpoles to be in close proximity with residual infection [38]. Tanks were monitored on a daily basis. Dead tadpoles were removed to prevent any scavenging, and stored at −25°C in individual plastic vials with ethanol for subsequent analyses.. The experiment terminated when all the individuals died or reached metamorphosis. The procedures used in this experiment follow protocol #2010-04-02 approved by the Laurentian University Animal Care Committee.

### Screening of tadpoles for FV3

In order to check for ranavirus infection, all animals (including euthanized ones) were dissected to remove the liver that was then crushed into a 1.5 ml Eppendorf tube. The resulting tissue mixture was used for DNA extraction. DNA was extracted using QIAmp DNeasy Kit following the standard protocol (Qiagen). After extraction, a double blind PCR was performed using a primer set known to successfully amplify a portion of the major capsid protein within the FV3 genome: MCP-ranavirus-F (5′-GACTTGGCCACTTATGAC-3′) and MCP-ranavirus-R (5′- GTCTCTGGAGAAGAAGAA), following the PCR conditions listed in Mao et al. [33], using 1.5 μl of template DNA and cycled 40 times. Individuals showing two positive amplifications for both PCRs were considered infected. We analyzed host survival using a survival analysis and failure time analysis following the Kaplan & Meier product limit method associated with Chi square and Gehan’s Wilcoxon tests (multiple and two sample comparisons respectively) [39]. Individuals surviving to the end of the experiment were censored to account for our lack of information about their true time of death [40].

### Viral DNA isolation

The SSME viral isolate was propagated on a confluent monolayer of A6 cells, and grown in a 75 cm^2^ flask, with cells infected at a multiplicity of infection (MOI) of 1 PFU/cell. The cells were harvested 5 days post infection (once cytopathic effect appeared), and viral DNA was extracted using the Purelink Viral RNA/DNA Mini Kit according to manufacturer’s protocol (Invitrogen, Burlington, ON).

### Viral genome sequencing

Standard kits and protocols developed by the manufacturer were used to sequence the SSME sample on a 454 GS-FLX platform (Roche Diagnostics Corporation). Briefly, a Rapid Library Preparation Kit (Roche, Mississauga, ON) was used to mechanically shear 500 ng of template DNA into short fragments. A universal sequencing primer that included a short DNA sequence unique to the sample (MID tag) was then annealed to both ends of each DNA fragment. A GS Junior Titanium Emulsion PCR Kit (Roche, Mississauga, ON) was used to amplify the sample library, which was sequenced using a GS Junior Titanium Sequencing Kit (Roche, Mississauga, ON). In order to assemble a full genomic sequence, the short sequences produced by 454 sequencing were aligned with the reference FV3 genome, wt-FV3, using GS Reference Mapper (Roche, Mississauga, ON). Any gaps in the assembled genome were then sequenced using custom PCR primers specific to each gap, with sequencing performed by the Robarts Sequencing Facility (London, ON). The final genomic sequence was deposited in GenBank accession number KJ175144.

### FV3 sample collection

FV3 environmental samples were collected from frogs caught by hand at various sites along a lakeshore in Manitoulin, Kagawong, ON, Canada (Latitude: 45.86418, Longitude: −82.27150). The frogs were caught using disposable gloves which were changed between each animal inspection. This method is preferred to the ‘net-catching method’ as it has been suggested that cross contamination can occur via the net. Each individual was toe clipped following the protocol #2009-03-04 approved by the Laurentian University Animal Care Committee for tissue sample collection. DNA was then isolated from toe clippings using the DNeasy Blood & Tissue Kit according to the manufacturer’s protocol for total DNA extraction from animal tissues (Qiagen, Mississauga, ON). Samples then underwent PCR with primers designed to amplify specific repeat regions identified in Eaton et al. [19]. Primers used included: Region 1-F: CGTGGTCAGACTGGTCCTCG; Region 1-R: CACCTCTGTCTCTGAATCGG; Region 2-F: GAGTTTACTTGGTGGCCATG; Region 2-R: TCCTGTCAAGAGATCCCCTC; Region 3-F: CTTGCTGCTGCCGTTCAGGC; and Region 3-R: AGAGTGAAAAAGGTAAAGGC.

### Sequencing repeats and confirming 454 sequence reads

In a PCR reaction tube the following reactants were combined: 10X PCR buffer (Invitrogen, Burlington, ON), 50 mM MgCl_2_ solution (Invitrogen, Burlington, ON), 5X TAQ DNA polymerase (Invitrogen, Burlington, ON), 10 mM deoxyribonucleotide triphosphates (dNTPs), 0.1 mM primer, 2.5 ng DNA, and water to a final volume of 50 μl. The reactions were then placed in a thermocycler under the following conditions: 94°C for 3 minutes, 94°C for 30 seconds, 56°C for 1 minute, and 72°C for 1 minute for 30 cycles. Sequences of PCR products were determined by Robarts Research Institute DNA Sequencing Facility in London, ON, and were analyzed using BioEdit v7.0.5.

## Competing interests

The authors declare that they have no competing interests.

## Authors’ contributions

EAM participated in the design of the study, isolated viral strains *in vitro*, carried out the molecular genetic studies, participated in 454 GS-FLX sequence alignment, and drafted the manuscript. SG participated in 454 GS-FLX sequence alignment. PE collected viral isolates from the field, carried out the immunoassays, and participated in the design of the study. DL participated in the design of the immunoassays. CJK oversaw the 454 GS-FLX sequencing. CRB conceived of the study, and participated in its design and coordination and helped to draft the manuscript. All authors have read and approved the final manuscript.
